# Transcriptome and proteome analyses reveal the potential mechanism of seed dormancy release in *Amomum tsaoko* during warm stratification

**DOI:** 10.1186/s12864-023-09202-x

**Published:** 2023-03-02

**Authors:** Chunliu Pan, Lixiang Yao, Liying Yu, Zhu Qiao, Meiqiong Tang, Fan Wei, Xueyan Huang, Yunyi Zhou

**Affiliations:** 1 Guangxi TCM Resources General Survey and Data Collection Key Laboratory, Guangxi Botanical Garden of Medicinal Plants, Nanning, China; 2 Guangxi Medicinal Resources Conservation and Genetic Improvement Key Laboratory, Guangxi Botanical Garden of Medicinal Plants, Nanning, China

**Keywords:** *A. tsaoko*, Physiological dormancy, Transcriptomic, Proteomic, Warm stratification, Molecular mechanism

## Abstract

**Background:**

In *Amomum tsaoko* breeding, the low germination rate is the major limitation for their large-scale reproduction. We found that warm stratification was an effective treatment to break the seed dormancy of *A. tsaoko* prior to sowing and could be an important component of improving breeding programs. The mechanism of seed dormancy release during warm stratification remains unclear. Therefore, we studied the differences between transcripts and proteomes at 0, 30, 60, and 90 days of warm stratification, to identify some regulatory genes and functional proteins that may cause seed dormancy release in *A. tsaoko* and reveal their regulatory mechanism.

**Results:**

RNA-seq was performed for the seed dormancy release process, and the number of differentially expressed genes (DEGs) was 3196 in three dormancy release periods. Using TMT-labelling quantitative proteome analysis, a total of 1414 proteins were defined as differentially expressed proteins (DEPs). Functional enrichment analyses revealed that the DEGs and DEPs were mainly involved in signal transduction pathways (MAPK signaling, hormone) and metabolism processes (cell wall, storage and energy reserves), suggesting that these differentially expressed genes and proteins are somehow involved in response to seed dormancy release process, including MAPK, PYR/PYL, PP2C, GID1, GH3, ARF, AUX/IAA, TPS, SPS, and SS. In addition, transcription factors ARF, bHLH, bZIP, MYB, SBP, and WRKY showed differential expression during the warm stratification stage, which may relate to dormancy release. Noteworthy, XTH, EXP, HSP and ASPG proteins may be involved in a complex network to regulate cell division and differentiation, chilling response and the seed germination status in *A. tsaoko* seed during warm stratification.

**Conclusion:**

Our transcriptomic and proteomic analysis highlighted specific genes and proteins that warrant further study in fully grasping the precise molecular mechanisms that control the seed dormancy and germination of *A. tsaoko*. A hypothetical model of the genetic regulatory network provides a theoretical basis for overcoming the physiological dormancy in *A. tsaoko* in the future.

**Supplementary Information:**

The online version contains supplementary material available at 10.1186/s12864-023-09202-x.

## Introduction

*Amomum tsaoko* Crevost et Lemarie (Zingiberaceae) is a traditional food and medicinal plant owing to its strong curative effects, such as drying dampness, stopping malaria, and eliminating phlegm, and its distinctive aroma of fruits [1]. As a perennial herb, *A. tsaoko* evolved dormancy to avoid injury in unsuitable environments, and this trait often occurs in seeds. It has been extensively cultivated in China as a high-value medicine plant in recent years. However, *A. tsaoko* seeds have characteristics of dormancy, which was caused by embryo physiological dormancy [2]. A high-quality seed with a high germination rate and rapid germination is a prerequisite for *A. tsaoko* production, and the seed dormancy release is directly related to breeding efficiency and affects the production of medicinal material. Therefore, it is of great significance to elucidate the mechanism of seed dormancy release of *A. tsaoko* for its standardized cultivation.

Seed dormancy is defined as the failure of an intact viable seed to germinate and establish seedling under favorable environmental conditions. Temperature is the main environmental factor that controls the seed dormancy release in physiological dormancy [3]. Warm or cold stratification has performed an important role during the seed dormancy release of several plants. For example, dormant seeds of *Arabidopsis* germinated to a high percentage after 5 days of stratification at 4 °C [4]. The stratification at 2 °C was more effective than at 5 °C in breaking seed dormancy of six populations of *Halenia elliptica*, and no dormancy break occurred at 8 °C [5]. Some studies suggest that the duration of stratification also affects seed dormancy release. The seed germination rate of pear rapidly increased and reached 85% after 50 days of cold stratification [6]. Moreover, a study of *Paris polyphylla* showed that seed dormancy release during warm stratification is controlled not only by the temperature but also by the duration of stratification [7], indicating that seed dormancy release is precisely regulated by temperature and time.

Massive genes and proteins have been identified as major factors contributing to seed dormancy maintenance or release. DOG1 (DELAY OF GERMINATION 1) is a major regulator of seed dormancy and the flowering process and has a synergistic effect with phytohormones. During *Arabidopsis* seed germination, *dog1* mutants exhibited increased germination by more than 60% in response to cold stratification [8]. Overexpression of DOG1 increased the expression level of the GA20ox gene in a temperature-dependent manner [9]. Abscisic acid (ABA) is the main plant hormone regulating seed dormancy and germination. Alternating temperatures caused a significant reduction in the expression of ABA-signaling genes in *Euphorbia esula* [10]. Moreover, alternating temperatures decreased ABA content and inhibited ABA biosynthesis key enzyme 9-cis-epoxycarotenoid dioxygenase (NCED) and ABA-responsive transcription factor gene ABSCISIC ACID INSENSITIVE5 (ABI5) to terminate dormancy [11]. In dormant *Arabidopsis* seeds, alternating temperatures can break dormancy by increasing the expression of GA3ox1 mediated through phytochrome B [12]. Meanwhile, the transcription factors (TFs) ERF, MYB, bHLH, NAC, WRKY, and MADS play important roles in the seed dormancy release of pear during cold stratification [6]. Additionally, the transcription factor ABA INSENSITIVE4 (ABI4) affects the seed dormancy status by targeting genes related to ABA and GA biosynthesis, catabolism, and signal transduction [13, 14]. The *Arabidopsis* B3-domain transcription factor FUSCA3 interacts with Sucrose Non-Fermenting-1-Related Kinase1 (AKIN10) and both act as positive regulators involved in ABA response [15]. Sucrose and starch are hydrolyzed into glucose by sucrose synthase (SS) and α-amylase (AMY) during the dormancy release in pear seeds and *PcaSS8* may play an important role in this process [16]. Transcriptomes and proteomes analyses can efficiently identify the genes and the proteins involved in hormone signaling, energy metabolism and mobilization of storage material during seed dormancy release in stratification, as reported in *Arabidopsis thaliana*, *Paris polyphylla*, *Pyrus calleryana*, and *Rosa canina* [4, 6, 7, 17]. Our previous study recognized the ABA biosynthesis gene (9-cis-epoxycarotenoid dioxygenase, NCED) as a negative regulator in seed dormancy release during warm stratification [18]. The expression patterns of ten reference genes (GAPDH, the 40 S, actin, tubulin, EIF4A-9, EIF2α, UBC, UBCE2, 60 S, and UBQ) at different dormancy release stages were identified [19]. However, the lack of research on the molecular biology of *A. tsaoko* limits an in-depth understanding of its mechanism of dormancy release.

The complex molecular networks that breaking of seed dormancy involve the integration of environmental cues and hormonal signals into regulatory mechanisms that include transcriptional, translational, and epigenetic processes [20, 21]. Although a variety of dormancy-release regulatory networks have been documented in plants [4, 6, 7, 17], relatively few investigations have attempted to characterize the mechanism concerning the dormancy release of *A. tsaoko* seeds. *A. tsaoko* seed dormancy was significantly broken in warm stratification. To explore the dormancy release molecular mechanism of *A. tsaoko* seeds, three different time categories of warm stratification seeds representing different degrees of dormancy release were studied. Transcriptome and proteome analysis was conducted and helped identify numerous differentially expressed genes and proteins at three different dormancy release periods. The results uncovered comprehensive expression pattern data for key genes and proteins during the dormancy release process in *A. tsaok*o seeds, which deepened our understanding of the molecular mechanisms of seed dormancy and germination in *A. tsaoko*.

## Results

### Effects of warm stratification on germination capacity, endogenous hormone content and microstructure of ***A. tsaoko*** seeds

**Fig. 1:**
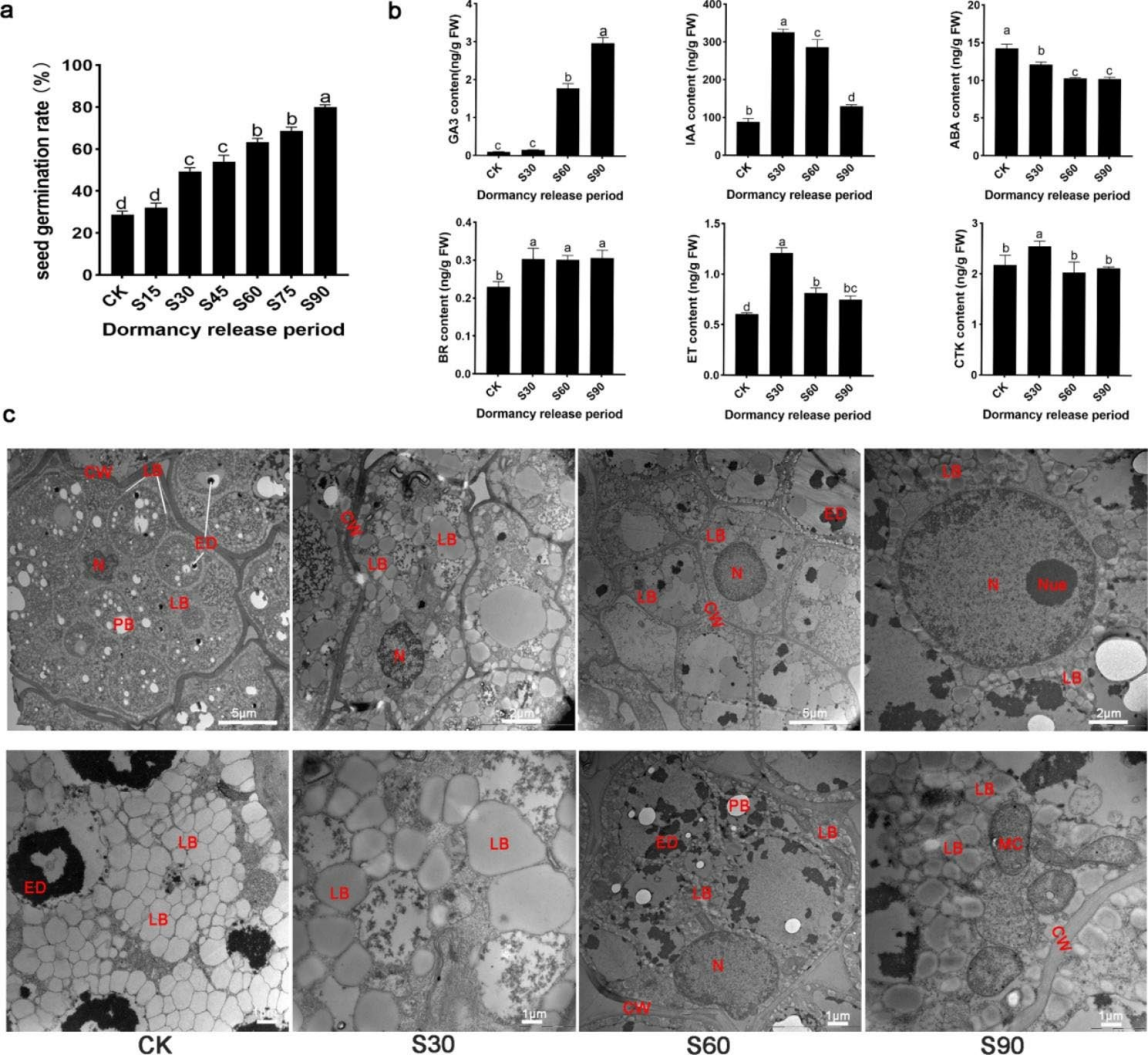
The effects of warm stratification treatment in *A. tsaoko* seeds during the dormancy release period. (a) Seed germination rate. (b) Endogenous hormones contents. (c) Electron microscopic micrographs of cross-sections of embryos. Abbreviations: CW, cell wall; PB, protein bodies; N, nucleus; Nue, nucleoli; LB, lipid bodies, ED, electron dense; MC, mitochondrion. CK, unstratified seeds; *A. tsaoko* seeds under warm stratification treatment by 15 days (S15), 30 days (S30), 45 days (S45), 60 days (S60), 75 days (S75) and 90 days (S90)

There was no remarkable change in morphology and size of seeds and embryos of *A.tsaoko* during stratification treatment by 15 days (S15), 30 days (S30), 45 days (S45), 60 days (S60), 75 days (S75) and 90 days (S90) (Fig. S1). But the germination rate of *A.tsaoko* increased significantly with the stratification time prolonged. The germination rate of unstratified seeds (CK) was only 28%, while it reached 52% at 30 days (S30) of stratification and the highest at 80% after 90 days (S90) of stratification (Fig. 1a). However, the germination rate of 15 days (S15), 45 days (S45), and 75 days (S75) exhibited no significant change with unstratified seeds (CK), 30 days (S30), and 60 days (S60), respectively. Thus, S30, S60 and S90 were defined as early, middle, and late periods of seed dormancy release, respectively.

Six classes of hormone including gibberellin (GA_3_), auxin (IAA), abscisic acid (ABA), brassinosteroid (BR), ethylene (ET) and cytokinin (CTK) in different stratification times were detected (Fig. 1b). The contents of GA_3_ did not fluctuate in unstratified seeds (CK) and the early period of seed dormancy release (S30), but showed a significant increase in the middle period of seed dormancy release (S60). The content of IAA increased first in the early period of seed dormancy release (S30) and then decreased in the late period of seed dormancy release (S90). The contents of ABA were high in unstratified seeds (CK), followed by a decrease after stratification. The content of BR slightly fluctuated during seed dormancy release. During seed dormancy release, the changing trend of ET and CTK content was similar to that of IAA content, with high content in the early period of seed dormancy release (S30). Overall, compared to unstratified seeds (CK), the GA_3_, IAA, BR, and ET contents of stratified *A. tsaoko* seeds were high, whereas the contents of ABA were low.

To further understand the features of seeds during dormancy release, transverse sections of embryos at different stratification stages were observed by transmission electron microscope (TEM) (Fig. 1c). In unstratified seeds (CK), a large number of lipid bodies were observed, exhibiting closely arrangement. After 30 days of stratification, lipid bodies were in a less tight arrangement, and the nuclei boundary was relatively clear. The nuclei were nearly round after 60 days of stratification, and lipid degradation occurred. Nucleoli and mitochondrion were found after 90 days of stratification. Overall, warm stratification treatment increased the germination rate, impacted hormone content, improved energy degradation, shortened physiological dormancy, and promoted seed dormancy release progression in *A. tsaoko*.

### Transcriptome data analysis

**Fig. 2:**
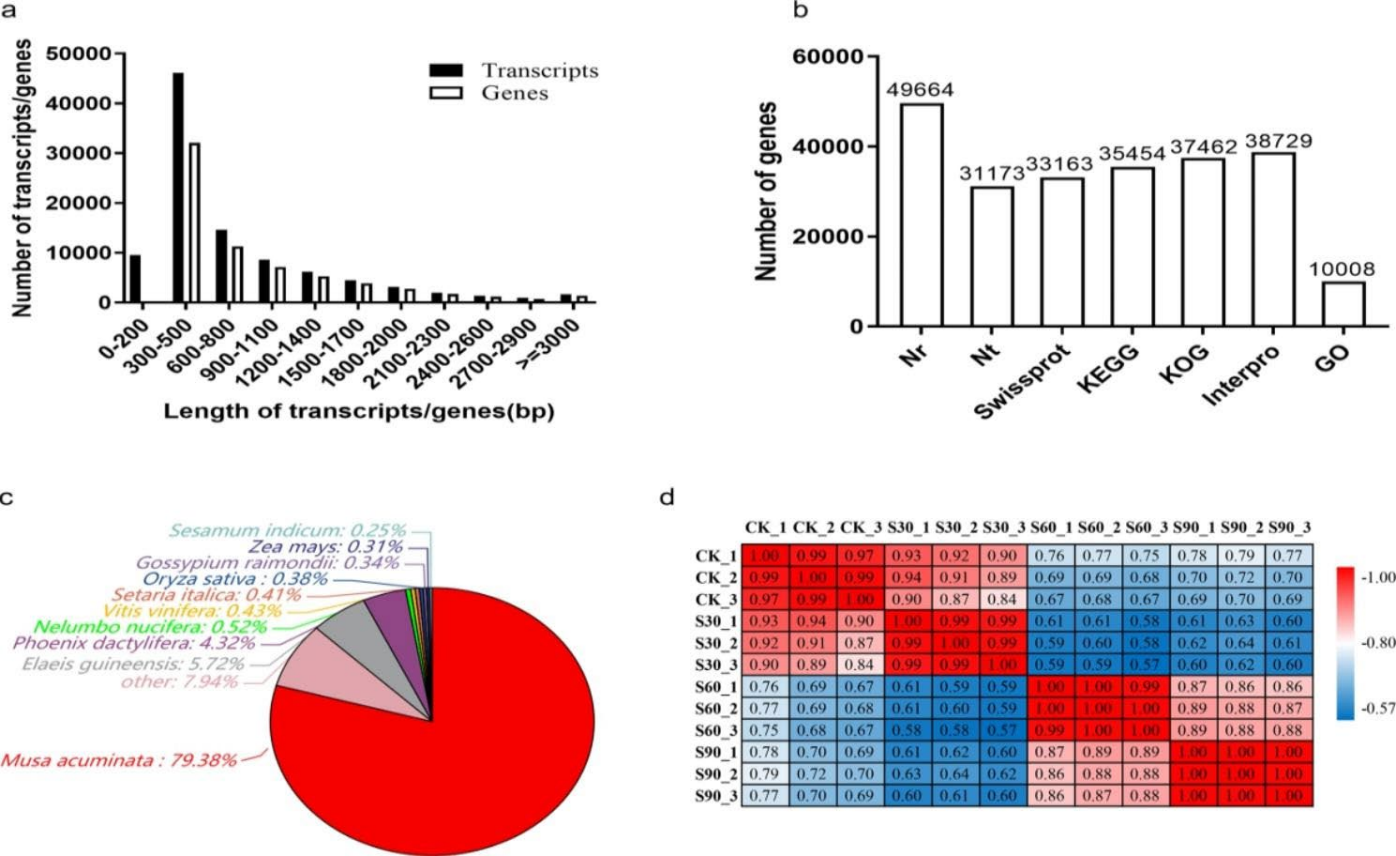
The annotation of *A. tsaoko* assembled transcriptome. (a) Length distribution of assembled *A. tsaoko* transcripts and genes. (b) Number of genes aligned to different databases. (c) Distribution of species aligned by assembled *A. tsaoko* genes. (d) Correlation analysis between samples replicates

To identify the regulation of gene expression during different seed dormancy release periods under warm stratification, the triplicate samples from the four different seed dormancy release periods were used to construct a total of 12 RNA-seq libraries. Meanwhile, the transcriptome library was constructed from a pool of mixed RNA consisting of the different seed dormancy release periods (named At_Mix). Approximately 87.14 Mb of original data in total was gained from the BGISEQ-500 platform at BGI-Shenzhen (Table S1). After removing low-quality reads and adapter sequences, 6.63 Gb clean reads were obtained and processed by de novo analysis using Trinity software. The assembly produced a total of 98,679 transcripts. Then, Tgicl software was used to perform clustering and eliminate redundant data in the transcripts, and 67,486 genes were gained. The N50 statistic was 1269, which meant that more than 50% of the genes were longer than 1269 bp. The length distribution of all the assembled genes was shown in Fig. 2a, which indicated that 6.08% of the complete transcripts and 7.57% of the total genes were longer than 2000 bp.

A total of 67,486 genes were functionally annotated with 7 functional databases (NR, NT, SwissProt, KEGG, KOG, InterPro and GO), 49,664 (73.59%), 31,173 (46.19%), 33,163 (49.14%), 35,454 (52.54%), 37,462 (55.51%), 38,729 (57.39%), 10,008 (14.83%) genes were annotated functionally respectively (Fig. 2b). 23,585 genes were commonly annotated in NR, KOG, KEGG, SwissProt and InterPro databases. Based on the functional annotation results of the NR database, the proportions of different species in the notes of genes were calculated, 39,423(79.38%) genes were aligned to *Musa acuminata* (Fig. 2c).

After removing low-quality reads and adapter sequences, 78.36 Mb clean reads were obtained, with a mean of 6.53 Gb clean reads for twelve RNA-Seq analysis libraries, and a total of 67,137 expressed genes were detected (Table S1). The average mapping rate of the transcriptome library (named At_Mix) was 83.62%. A heat map cluster showed high correlations among each replicate which indicated good repeatability of the data (Fig. 2d).

### GO term and KEGG pathway identification

**Fig. 3:**
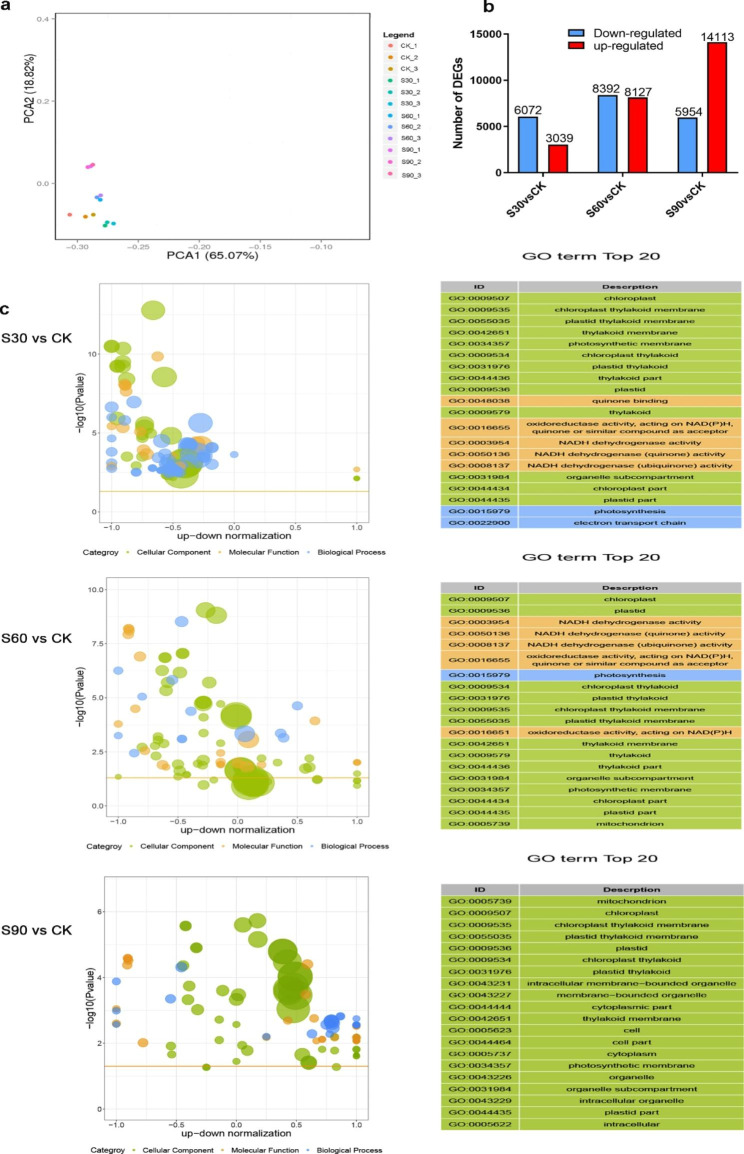
Genes detected and expressed in seed dormancy during the different stratification periods. (a) Principal component analysis of the RNA-Seq data. (b) The numbers of up-regulated and down-regulated genes. (c) Gene enrichment term assignments for the DEGs

It was noted that the samples from the different seed dormancy release periods could be clustered into four groups by using a principal component analysis (Fig. 3a). The number of DEGs between the S90 vs. CK was the largest, followed by the S60 vs. CK, and the fewest was found in comparisons between the S30 and CK (Fig. 3b).

GO enrichment analysis was performed to further assess the functions of the DEGs across the different seed dormancy release periods (Fig. 3c). The top 20 significantly enriched GO terms concerning cellular component (CC), molecular function (MF), and biological process (BP) were shown in the three comparisons. The chloroplast, chloroplast thylakoid membrane, plastid thylakoid membrane, thylakoid membrane and photosynthetic membrane were identified as enriched GO terms in S30 vs. CK group. The chloroplast, plastid, NADH dehydrogenase activity, NADH dehydrogenase (quinone) activity and NADH dehydrogenase (ubiquinone) activity were identified as enriched GO terms in S60 vs. CK group. The mitochondrion, chloroplast, chloroplast thylakoid membrane, plastid thylakoid membrane and plastid were identified as enriched GO terms in S90 vs. CK group. GO enrichment analysis indicated genes associated with the chloroplast, plastid, thylakoid membrane and NADH dehydrogenase activity may involve in the regulation of seed dormancy release during warm stratification.

**Fig. 4:**
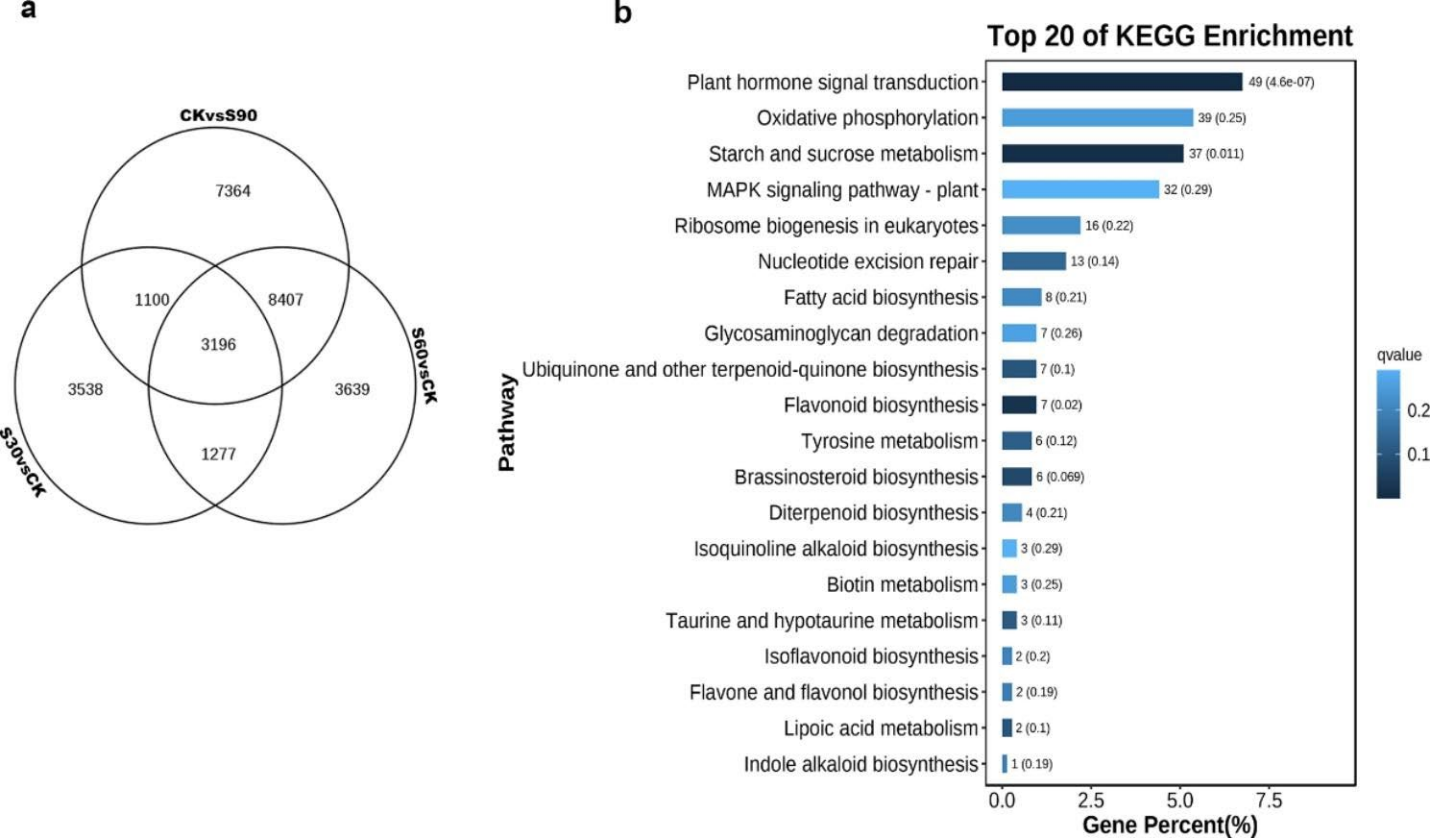
Overview of the Venn diagram and KEGG enrichment annotation. (a) Venn diagram showing the overlap between the total number of DEGs identified in each of the three comparisons. (b) KEGG enrichment annotation of the 3196 DEGs shared by each of the three comparisons

Besides, the results also revealed several significant differentially expressed genes involved in embryo development ending in seed dormancy and seed germination (Table 1). Venn diagram showed that 3196 differentially expressed genes were shared in all three comparisons (S30 vs. CK, S60 vs. CK and S90 vs. CK) (Fig. 4a and Table S2), implying that they may play an important role in the process of seed dormancy release by warm stratification treatment. Classification of 3196 genes using KEGG pathway analysis was performed to evaluate the potential functions of these DEGs in seed dormancy release periods. It is worth noting that plant hormone signal transduction, oxidative phosphorylation, starch and sucrose metabolism, and MAPK signaling pathway were most significantly enriched (Fig. 4b). Therefore, changes in phytohormones, energy release, energy metabolism and signaling cascades may be an important event in seed dormancy release of *A.tsaoko*.

**Table 1:** Functional classification and pathway assignment of seed dormancy/germination-related genes by GO

GO ID	Gene ID	log2Ratio(S30/CK)	log2Ratio(S60/CK)	log2Ratio(S90/CK)
**GO:0009793 embryo development ending in seed dormancy**				
Transcription factor HBP-1b(c38)-like	Unigene35675_At_Mix	-1.54037	-1.93607	-1.30578
Auxin-repressed 12.5 kDa protein-like	Unigene933_At_Mix	1.530515	2.485427	2.751321
Transcription initiation factor TFIID	CL4011.Contig1_At_Mix	0.579668	0.271378	1.622061
Peptidyl-prolyl cis-trans isomerase PASTICCINO1	CL7831.Contig2_At_Mix	0.635844	1.547488	2.086157
Leucine–tRNA ligase	Unigene12495_At_Mix	1.521397	-0.95109	1.572472
Leucine–tRNA ligase	Unigene15613_At_Mix	0.817136	1.947533	2.918295
AMP deaminase-like	Unigene22288_At_Mix	-5.24793	1.658963	2.484523
Glutamate carboxypeptidase 2	Unigene27835_At_Mix	-0.45519	0.491476	2.086464
SKP1-like protein 1B	Unigene39191_At_Mix	-0.54946	-0.9446	1.606198
**GO:0009845 seed germination**				
GEM-like protein 5	CL3614.Contig1_At_Mix	-0.25211	-0.868	-1.31374
Polypyrimidine tract-binding protein homolog 1-like	Unigene14359_At_Mix	1.354431	-6.30378	1.111257

### Identification and functional classification of differentially expressed genes

To further elucidate the role of these intracellular events in the process of seed dormancy release with 3196 genes, the MAPK signaling pathway, plant hormone signal transduction, starch and sucrose metabolism and fatty acid biosynthesis were selected for analysis (Table S3, Fig. S2). 20 DEGs were identified to be highly similar to the MAPK signaling pathway and were observed as differentially regulated during the process of seed dormancy release by warm stratification treatment (Fig. S2a). Among them, 16 DEGs observed a decreasing trend during seed dormancy release progression, including 9 serine/threonine protein kinases genes (MAPKKK, MAPKK, and MAPK), 5 LRR receptor-like serine/threonine-protein kinase genes (LRR-RLKs) and 2 protein kinase (PK). A total of 27 DEGs were identified to be highly similar to many plant hormone signal pathways. Auxin (IAA) and abscisic acid (ABA) are major constituents of plant hormone signal pathways during the process of seed dormancy release by warm stratification treatment (Fig. S2b), including 3 auxin response factor (ARF), 3 auxin-responsive protein (AUX/IAA), 2 indole-3-acetic acid-amido-synthetasesase (GH3), 4 abscisic acid receptor (PYR/PYL), 3 protein phosphatase 2 C (PP2C) and 1 abscisic acid-insensitive protein (ABF). Interestingly, AUX/IAA genes (Unigene38811_At_Mix, CL1033Contig3_At_Mix and Unigene38530_At_Mix) exhibited an increasing trend during the process of seed dormancy release. 34 DEGs were identified to be highly similar to the starch and sucrose metabolism and were observed as differentially regulated during the process of seed dormancy release by warm stratification treatment (Fig. S2c). The major constituents of starch and sucrose metabolism during the process of seed dormancy release are sucrose phosphate synthase genes (SPS), starch synthase (SS), and beta-amylase, alpha-amylase, alpha,alpha-trehalose-phosphate synthase(TPS), trehalose-phosphate phosphatase (TPP), alpha-glucosidase, endoglucanase, alpha-glucan phosphorylase (PHS) and glucose-1-phosphate adenylyltransferase (AGPS). Among them, all of SPS (Unigene39974_At_Mix, Unigene11451_At_Mix and Unigene14649_At_Mix), alpha-glucosidase (Unigene14905_At_Mix and Unigene32157_At_Mix), TPS (CL33.Contig1_At_Mix and CL6551.Contig2_At_Mix), alpha-glucosidase (Unigene14905_At_Mix and Unigene32157_At_Mix) and beta-amylase (CL2385.Contig3_At_Mix and CL2385.Contig4_At_Mix) genes exhibited decreasing trend, while AGPS (Unigene27153_At_Mix, Unigene39263_At_Mix and CL2759.Contig1_At_Mix) and alpha-amylase (CL5401.Contig2_At_Mix) genes exhibited increasing trend during the process of seed dormancy release. A total of 7 genes homologous to the genes associated with fatty acid biosynthesis were observed as differentially regulated during the process of seed dormancy release (Fig. S2d). Meanwhile, palmitoyl-acyl carrier protein thioesterase (CL2315.Contig1_At_Mix and CL2315.Contig4_At_Mix) exhibited an increased trend in expression in the release of seed dormancy (Fig. 4d).

Notably, the genes identified in the present study mapped to the transcription factors, cell wall and heat shock protein. More specifically, 23 genes belonging to 10 TF families were differentially expressed during the process of seed dormancy release (Fig. S3). bHLH, WRKY, and BES1 were enriched (Fig. S3a). A total of 9 transcripts homologous to the genes associated with cell wall were observed as differentially regulated during the process of seed dormancy release including xyloglucan endotransglucosylase/hydrolase (XTH) and expansin genes (Fig. S3b). Meanwhile, most of the XTH and expansin genes exhibited decreased trend in expression in the release of seed dormancy. 17 with heat shock protein were differentially expressed during the process of seed dormancy release (Fig. S3c). Interestingly, the CL5269.Contig2_At_Mix was identified as a heat stress transcription factor and exhibited a decreasing trend during the seed dormancy release period.

### Proteome sequencing analysis

**Fig. 5:**
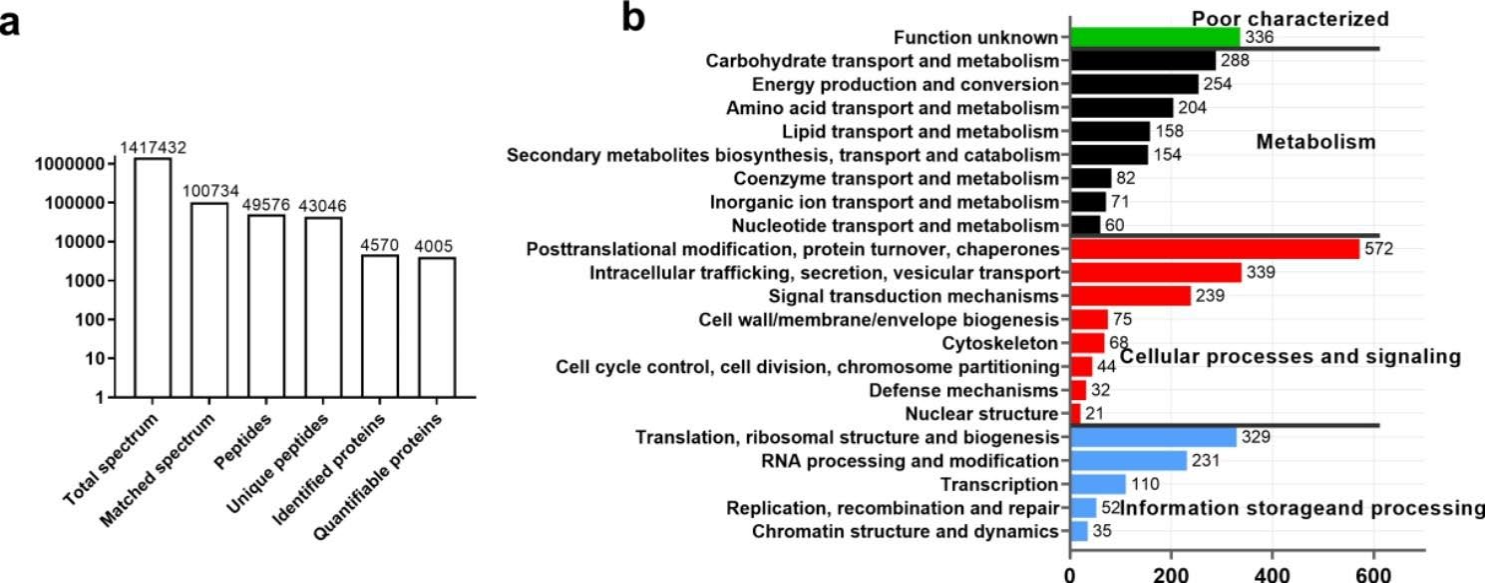
Overview of the *A. tsaoko* proteome. (a) The numbers of tandem mass spectrometry spectra and the number of proteins and unique peptides identified by the proteome analysis. (b) KOG annotation of the *A. tsaoko* proteins identified

A total of 1,417,432 spectrums were yielded in proteome sequencing analysis, of which 43,046 unique peptides were obtained. After removing the peptides with an FDR (false discovery rate) value > 1%, a total of 4570 proteins was identified (Fig. 5a). Based on the KOG analysis (Fig. 5b), most proteins were found in the cellular processes and signaling categories including posttranslational modification, protein turnover, chaperones and intracellular trafficking, secretion, vesicular transport, followed by the metabolism category involved in carbohydrate transport and metabolism, energy production and conversion and amino acid transport and metabolism. In addition, 329 proteins were grouped into translation, ribosomal structure and biogenesis.

### Identification and functional annotation of differentially expressed proteins

**Fig. 6:**
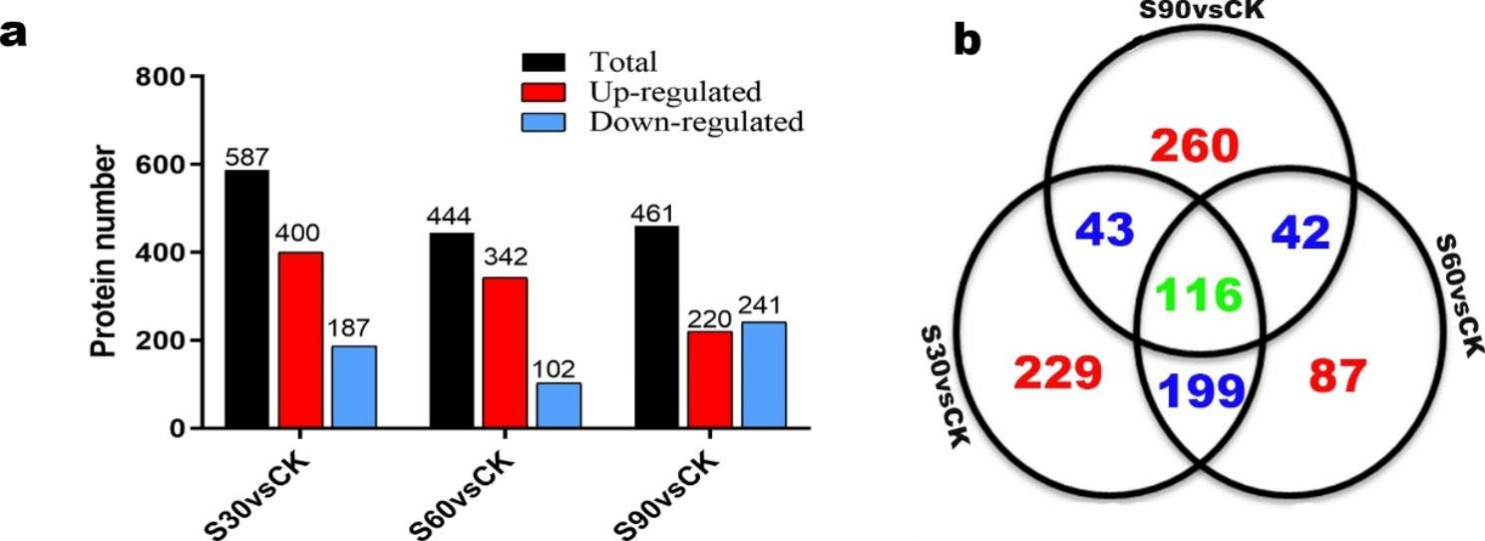
Overview of the number of DEPs number and fold changes in their expression levels in three comparisons. (a) Number of upregulation and downregulation proteins for each of the three comparisons. (b) Venn diagram showing the overlap between the total number of DEPs identified in each of the three comparisons

1414 proteins were differentially expressed at one or more of the three time points (Table S4). Classification of DEPs using GO and KEGG pathway analysis was performed to evaluate the potential functions of these DEPs (Table S5 and Fig. S4). In the S30 vs. CK comparison, 587 proteins were significantly differentially expressed, of which 400 were up-regulated and 187 were down-regulated (Fig. 6a). In the S60 vs. CK, S90 vs. CK comparisons, 342 and 220 proteins were significantly up-regulated, respectively, and 102 and 241 were significantly down-regulated, respectively. A total of 116 proteins were common among the three comparisons (S30 vs. CK, S60 vs. CK and S90 vs. CK) (Fig. 6b), indicating a certain similarity in changes of protein at different time points of seed dormancy release.

Based on the result of the GO and KEGG pathway analysis for 116 common proteins, the pathways were as follows: MAPK signaling pathway, plant hormone synthesis and signal transduction, starch and sucrose metabolism, glycolysis/gluconeogenesis, fatty acid metabolism, transcription factors, cell wall proteins, defense response, seed dormancy and germination proteins (Table 2). MAPK3 (mitogen-activated protein kinase 3) was up-regulated during seed dormancy release periods in the MAPK signaling pathway. GA2OX8 (gibberellin 2-beta-dioxygenase 8) was down-regulated, GH3.5 (indole-3-acetic acid-amido synthetase GH3.5), and PYR1 (abscisic acid receptor PYR1) were up-regulated during seed dormancy release periods in plant hormone synthesis and signal transduction pathways. BGLU6 (beta-glucosidase 6) was up-regulated during seed dormancy release periods, and WAXY (amyloplastic) and SS2 (sucrose synthase 2) were up-regulated in the early and middle phase of seed dormancy release but down-regulated later in starch and sucrose metabolism pathways. Two PDC2 (pyruvate decarboxylase 2) and one PLDBETA1 (phospholipase D beta 1) protein were up-regulated during seed dormancy release periods. Transcription factor MYB44 was up-regulated, and AIL5 (AP2-like ethylene-responsive transcription factor) was down-regulated during seed dormancy release periods. XTH (xyloglucan endotransglycosylases/hydrolases) and EXPA4 (expansin-A4) proteins were up-regulated during seed dormancy release periods. Heat shock proteins HSP81 and HSP82 were down-regulated in the late period of seed dormancy release. Two proteins associated with seed dormancy and germination were up-regulated in the middle and late seed dormancy release periods.

**Table 2:** Differentially expressed proteins (DEPs) identified during the seed dormancy breaking period by warm stratification treatment

	Protein accession	Gene name	S30/CK Regulated Type	S60/CK Regulated Type	S90/CK Regulated Type
**MAPK signaling pathway**					
Mitogen-activated protein kinase 3	Gene.43702_Unigene6721_At_Mix	MAPK3	Up	Up	Up
**Plant hormone synthesis and signal transduction**					
Gibberellin 2-beta-dioxygenase 8	Gene.54345_Unigene23473_At_Mix	GA2OX8	Down	Down	Down
Indole-3-acetic acid-amido synthetase GH3.5	Gene.19270_CL4276.Contig5_At_Mix	GH3.5	Up	Up	Up
Protein phosphatase 2 C	Gene.1760_CL332.Contig4_At_Mix	PP2C	Down	Down	Up
Abscisic acid receptor PYR1	Gene.36366_CL9730.Contig1_At_Mix	PYR1	Up	Up	Up
**Starch and sucrose metabolism**					
Beta-glucosidase 6	Gene.29384_CL7237.Contig5_At_Mix	BGLU6	Up	Up	Up
Amyloplastic	Gene.62705_Unigene35857_At_Mix	WAXY	Up	Up	Down
Sucrose synthase 2	Gene.43938_Unigene6964_At_Mix	SS2	Up	Up	Down
**Glycolysis / Gluconeogenesis**					
Pyruvate decarboxylase 2	Gene.22279_CL5048.Contig4_At_Mix	PDC2	Up	Up	Up
Pyruvate decarboxylase 2	Gene.22274_CL5048.Contig2_At_Mix	PDC2	Up	Up	Up
**Fatty acid metabolism**					
Phospholipase D beta 1	Gene.18698_CL4123.Contig2_At_Mix	PLDBETA1	Up	Up	Up
**Transcription factors**					
Transcription factor MYB44	Gene.43178_Unigene6226_At_Mix	MYB44	Up	Up	Up
AP2-like ethylene-responsive transcription factor	Gene.7503_CL1582.Contig1_At_Mix	AIL5	Down	Down	Down
**Cell wall protein**					
Xyloglucan endotransglycosylases/hydrolases	Gene.36618_CL9824.Contig1_At_Mix	XTH7	Up	Up	Up
Expansin-A4	Gene.9479_CL1996.Contig3_At_Mix	EXPA4	Up	Up	Up
**Heat shock protein**					
Heat shock protein 81	Gene.10515_CL2198.Contig1_At_Mix	HSP81	Up	Up	Down
Heat shock protein 82	Gene.34182_CL8907.Contig2_At_Mix	HSP82	Up	Up	Down
**Seed dormancy and germination protein**					
ASPARTIC PROTEASE IN GUARD CELL 2	Gene.49363_Unigene15486_At_Mix	ASPG2	Up	Up	Up
ASPARTIC PROTEASE IN GUARD CELL 2	Gene.43007_Unigene6063_At_Mix	ASPG2	Down	Up	Up

### Correlation analysis between differentially expressed genes and proteins

A correlation analysis between the transcriptome and proteome was performed to better understand the molecular mechanisms involved in the release of seed dormancy. In this study, 67,137 genes and 4570 proteins in transcriptome and proteome datasets were used for correlation analysis and the expression correspondence of 2364 transcripts and proteins was established (Table S6). Pearson’s correlation coefficient analysis showed a 45.9% positive correlation between transcriptome and proteome data, indicating a relatively obvious positive relationship between mRNA and protein expression overall (Fig. S5). The number of differentially expressed genes and proteins correlated were as follows: 209 for S30 vs. CK, 472 for S60 vs. CK and 655 for S90 vs. CK (Table 3). Among them, plant hormone signal transduction-related, starch and sucrose metabolism-related, glycolysis /gluconeogenesis-related, citrate cycle (TCA cycle)-related, fatty acid metabolism-related, transcription factors-related, cell wall-related, heat shock-related, seed dormancy and germination-related genes/proteins were identified (Table 4). In plant hormone signal transduction, GH3.5 exhibited a positively correlated expression between the transcriptome and proteome. TPS (alpha,alpha-trehalose-phosphate synthase) exhibited a positive correlation in starch and sucrose metabolism, and WAXY, SPS1(sucrose-phosphate synthase 1), and SS2 exhibited negatively correlated expression between the transcriptome and proteome. ENO1 (enolase 1) and ALDH2B7 (aldehyde dehydrogenase family 2 member B) exhibited a positive correlation in glycolysis/gluconeogenesis, and PDC2, PKP2 (plastidial pyruvate kinase 2), and ACS (glyoxysomal) exhibited negatively correlated expression. MMDH (malate dehydrogenase) and ACLA-3 (ATP-citrate synthase alpha chain protein 3) exhibited positively correlated expression in the citrate cycle. A total of five key genes and proteins involved in fatty acid metabolism were identified in the negative correlation analysis between the transcriptome and proteome. Transcription termination factor mTERF (mitochondrial transcription termination factors) was identified in the negative correlation analysis, and U2AF35B (splicing factor U2af small subunit B), GRP2 (glycine-rich protein 2), and C2H2 (zinc finger protein) exhibited positively correlated expression. Two expansin proteins exhibited positively correlated expression and six heat shock proteins exhibited negatively correlated expression between the transcriptome and proteome. Three proteins involved in seed dormancy and germination were identified in the transcriptome and proteome. Interestingly, two of three seed dormancy and germination proteins exhibited negatively correlated expression.

**Table 3:** Correlation analysis of differentially expressed genes and proteins identified from transcriptomic and proteomic analyses

	Gene number	Protein number	Correlation number
**S30vsCK**	9111	587	209
**S60vsCK**	16,519	444	472
**S90vsCK**	20,067	461	655

**Table 4:** Correlated differentially expression transcripts/proteins for the comparison

	Gene name	Expression pattern	Transcript.ID	Protein.ID
**Plant hormone signal transduction**				
Indole-3-acetic acid-amido synthetase GH3.5	GH3.5	positive	CL4276.Contig5_At_Mix	Gene.19270_CL4276.Contig5_At_Mix
**Starch and sucrose metabolism**				
Amyloplastic	WAXY	negative	Unigene35857_At_Mix	Gene.62705_Unigene35857_At_Mix
Alpha,alpha-trehalose-phosphate synthase	TPS	positive	CL4726.Contig3_At_Mix	Gene.21036_CL4726.Contig3_At_Mix
Sucrose-phosphate synthase 1	SPS1	negative	Unigene6303_At_Mix	Gene.43277_Unigene6303_At_Mix
Sucrose synthase 2	SS2	negative	Unigene6964_At_Mix	Gene.43938_Unigene6964_At_Mix
**Glycolysis / Gluconeogenesis**				
Pyruvate decarboxylase 2	PDC2	negative	CL5048.Contig2_At_Mix	Gene.22274_CL5048.Contig2_At_Mix
Enolase 1	ENO1	positive	Unigene1780_At_Mix	Gene.38591_Unigene1780_At_Mix
Aldehyde dehydrogenase family 2 member B	ALDH2B7	positive	Unigene26001_At_Mix	Gene.56044_Unigene26001_At_Mix
Plastidial pyruvate kinase 2	PKP2	negative	CL5395.Contig2_At_Mix	Gene.23557_CL5395.Contig2_At_Mix
glyoxysomal	ACS	negative	CL4713.Contig1_At_Mix	Gene.20984_CL4713.Contig1_At_Mix
**Citrate cycle (TCA cycle)**				
Malate dehydrogenase	MMDH	positive	Unigene27705_At_Mix	Gene.57253_Unigene27705_At_Mix
ATP-citrate synthase alpha chain protein 3	ACLA-3	positive	Unigene16500_At_Mix	Gene.50017_Unigene16500_At_Mix
**Fatty acid metabolism**				
2-methylacyl-CoA dehydrogenase	2MBCD	negative	Unigene13091_At_Mix	Gene.47938_Unigene13091_At_Mix
Long chain acyl-CoA synthetase 4	LACS4	negative	CL8918.Contig2_At_Mix	Gene.34208_CL8918.Contig2_At_Mix
3-ketoacyl-CoA thiolase 2	PED1	negative	CL1166.Contig2_At_Mix	Gene.5647_CL1166.Contig2_At_Mix
Peroxisomal acyl-coenzyme A oxidase 1	ACX1	negative	CL2195.Contig2_At_Mix	Gene.10506_CL2195.Contig2_At_Mix
Peroxisomal fatty acid beta-oxidation multifunctional protein	MFP	negative	Unigene31449_At_Mix	Gene.59650_Unigene31449_At_Mix
**Transcription factors**				
Splicing factor U2af small subunit B	U2AF35B	positive	CL4942.Contig6_At_Mix	Gene.21902_CL4942.Contig6_At_Mix
Glycine-rich protein 2	GRP2	positive	Unigene33171_At_Mix	Gene.60880_Unigene33171_At_Mix
Mitochondrial transcription termination factors	mTERF	negative	Unigene8826_At_Mix	Gene.45456_Unigene8826_At_Mix
Zinc finger protein	C2H2	positive	Unigene27716_At_Mix	Gene.57265_Unigene27716_At_Mix
**Cell wall protein**				
Expansin-A4	EXPA4	positive	CL4237.Contig1_At_Mix	Gene.19114_CL4237.Contig1_At_Mix
Expansin-A4	EXPA4	positive	CL7306.Contig1_At_Mix	Gene.29591_CL7306.Contig1_At_Mix
**Heat shock protein**				
Heat shock protein 81 − 1	HSP81-1	negative	CL2198.Contig1_At_Mix	Gene.10515_CL2198.Contig1_At_Mix
Heat shock protein 81 − 1	HSP81-1	negative	CL2198.Contig2_At_Mix	Gene.10518_CL2198.Contig2_At_Mix
Heat shock protein 81 − 1	HSP81-1	negative	CL2198.Contig3_At_Mix	Gene.10521_CL2198.Contig3_At_Mix
Heat shock protein 81 − 1	HSP81-1	negative	Unigene2709_At_Mix	Gene.39539_Unigene2709_At_Mix
Heat shock protein 81 − 1	HSP81-1	negative	Unigene6699_At_Mix	Gene.43667_Unigene6699_At_Mix
Heat shock protein 82	HSP82	negative	CL8907.Contig2_At_Mix	Gene.34182_CL8907.Contig2_At_Mix
**Seed dormancy and germination proteins**				
ASPARTIC PROTEASE IN GUARD CELL 1	ASPG1	negative	Unigene27450_At_Mix	Gene.57088_Unigene27450_At_Mix
ASPARTIC PROTEASE IN GUARD CELL 1	ASPG1	negative	CL1058.Contig2_At_Mix	Gene.5014_CL1058.Contig2_At_Mix
ASPARTIC PROTEASE IN GUARD CELL 2	ASPG2	positive	CL2169.Contig1_At_Mix	Gene.10399_CL2169.Contig1_At_Mix

### Validation of RNA-Seq by qRT-PCR analysis

The expression levels of 50 genes were used to verify the reliability of the transcriptome data. The expression trends of 42 genes (84.0%) during the seed dormancy release period by warm stratification were consistent with the transcriptome data, with only the absolute fold changes in gene expression showing slight differences (Table S7). XTH23 (Unigene36782_At_Mix), and beta-amylase 1 (CL2385.Contig3_At_Mix) were up-regulated expressed by qRT-PCR, but were down-regulated in the transcriptome analysis. MAPK (Unigene18236_At_Mix), SCL8 (CL3706.Contig2_At_Mix) and GH3 (Unigene3359_At_Mix) were up-regulated expressed in the middle and late period of seed dormancy release by qRT-PCR, but were down-regulated in the transcriptome analysis. NAC (CL36.Contig2_At_Mix), was up-regulated expressed in the late period of seed dormancy release by qRT-PCR, but was down-regulated in the transcriptome analysis. HSP (CL7421.Contig1_At_Mix), PP2C (CL1836.Contig3_At_Mix) and BAK1 (Unigene22932_At_Mix) were up-regulated expressed in the early and middle period of seed dormancy release by qRT-PCR, but was down-regulated in the transcriptome analysis. The correlation between “Fold change in qRT-PCR” and “Fold change in transcriptome” is moderately positive (R^2^ = 0.70) with a significance level of P<0.01 (Fig. S6).

## Discussion

Seed dormancy acts as the key regulated germination step during variable environmental conditions. The influence of temperature on seed dormancy status underlines the need to understand how changing environmental conditions will affect seed germination patterns. In recent studies, the temperature conditions, such as warm and cold stratification, affect dormancy and germination. However, the mechanism of seed dormancy release under special temperature conditions has not been entirely understood. The type of seed dormancy in *A. tsaoko* was physiological dormancy, and warm stratification was the optimal manner to break the seed dormancy of *A. tsaoko* [2]. Although stratification had a positive effect on seed dormancy release in *A. tsaoko*, the genes and proteins involved in the seed dormancy release process and their relationship are unclear. In the current study, to elucidate the molecular response of seeds during dormancy release, transcriptome and proteome technologies were conducted to investigate the common response mechanism of *A. tsaoko* seeds under warm stratification. Under warm stratification, 3196 genes and 1414 proteins were differentially regulated. The results showed that the MAPK signaling pathway, plant hormone signal transduction, starch and sucrose metabolism, glycolysis /gluconeogenesis, citrate cycle (TCA cycle), fatty acid metabolism, transcription factors, cell wall proteins, heat shock proteins, seed dormancy and germination proteins involved in the process of seed dormancy release.

## MAPK signaling cascade in ***A. tsaoko*** seed dormancy release progression

Mitogen-activated protein kinase (MAPK) pathways, being composed of three serine/threonine protein kinases (MAPKKK, MAPKK and MAPK), can transduce extracellular signals to regulate transcriptional events and other cellular processes. MAPK proteins have been reported to be involved in the response to ABA to control seed dormancy and germination. MKKK62 negatively controls rice seed dormancy through the MKKK62-MKK3-MAPK7/MAPK14 system and is involved in regulating the transcription of MFT (MOTHER OF FT AND TFL), and the overexpressing lines of MKKK62 in rice showed ABA sensitivity during the germination stage and the late stage of seed maturation [22]. Overexpressing MAP3K17 in *Arabidopsis* showed lower sensitivity to ABA during post-germinated growth, suggesting the negative roles of MAP3K17 in the response to ABA [23]. The expression of ABA biosynthesis gens NCED in *A. tsaoko* decreased gradually with the increase of stratification time [18], implying that there might be an ABA-responsive MAPK signaling cascade during seed dormancy release. A total of 21 genes encoding serine/threonine protein kinases were identified in the transcriptomic and proteomic data (Fig. S2a and Table 2). Among them, Unigene32911_At_Mix (MAPK3), Unigene33589_At_Mix (MAPK5) and Unigene18236_At_Mix (MAPKA) showed obvious down-regulated expression during the seed dormancy release period by warm stratification.

## Metabolism and regulation of plant hormone in ***A. tsaoko*** seed dormancy release progression

Plant hormones play important role in seed dormancy maintenance and release. In hormone assays, compared to unstratified seeds (CK), the GA_3_, IAA, BR, and ET contents of stratified A. tsaoko seeds were high, whereas the contents of ABA were low (Fig. 1b). Transcriptomic and protein database revealed some genes that are involved in ABA, GA, IAA, brassinosteroid (BR), cytokinin (CTK) and ethylene (ET) signaling pathways. In *Paris polyphylla* seeds, the metabolic and signaling genes related to ABA, GA, IAA, BR, CTK, ethylene, jasmonic acid (JA) and salicylic acid are differentially expressed among seed dormancy release during a warm stratification [7]. The PYL family proteins are ABA receptors, and PP2C proteins are negative regulators in the ABA signaling pathway. *PtPYRL1* and *PtPYRL5* positively regulated the ABA responses during the seed germination, associated with inhibiting type 2 C protein phosphatase (PP2C) activity [24]. The over-expression or loss of function of the PP2C proteins significantly altered seed dormancy in *Arabidopsis* and wheat [25–27]. GA acts through the GID1-DELLA-SCFSLY1/GID2 signaling cascade to regulate seed germination, whereas the GID1 receptor family and F-box protein gene GID2 are positive regulators [28, 29]. The expression level of two AUX1/IAA genes, six ARF genes and three GH3 genes were higher in germination seeds than in dormant seeds [7]. Overexpressing BRI in seeds was more rapidly germinated than wild type in the absence of cold stratification, suggesting BRI1 plays a role in seed germination and the release of dormancy [30]. Among hormone signal-related genes and proteins, PYR/PYL, PP2C, GID1, GID2, GH3, ARF AUX/IAA, BRI and BSK were differentially expressed in transcriptome and proteome during the seed dormancy release period by warm stratification (Fig. S2b, Tables 2 and 4). Overall, Consistent with hormone levels, the corresponding hormone signal-related genes were significantly induced during seed warm stratification, which indicates that hormones may play a role in regulating the seed dormancy release of *A. tsaoko*. Previous studies reveal that the crosstalk between ABA and other hormones such as GA, IAA, CTK and BR also play critical roles in seed dormancy, and germination [31]. The DELLA proteins, well-known negative GA signaling components, influence seed dormancy and germination [32, 33], by stimulating the ABA biosynthesis and ABI5 activity, in which DELLA promotes the expression of ABI5 and enhances the ABA-mediated repression of seed germination [34]. ARF mediates ABI3 activation in cross-talk with IAA and promotes seed dormancy but inhibits seed germination [35, 36]. ABI4 negatively regulated the transcription of ARR6, ARR7 and ARR15, revealing crosstalk between ABA and cytokinin signaling to inhibit seed germination [37]. In this study, ABA signaling-related genes in stratified seeds coordinately regulated other hormone genes by altering their expression levels. Overall, our results provide evidence that the dormancy release process of *A. tsaoko* seeds during a warm stratification is tightly correlated with hormone-signaling genes.

## Metabolism and regulation of storage and energy reserves in ***A. tsaoko*** seed dormancy release progression

Large numbers of genes and proteins, such as SPS, SUS, SS and SBE (starch branching enzyme), which are related to starch and sucrose metabolism pathways, were significantly up-regulated with the release of seed dormancy in Callery pear during cold stratification, and starch and soluble sugars content was in accord with the trend of genes expression [6]. Additionally, carrot seed transcriptome results suggested that the TCA cycle and glycolysis/gluconeogenesis are crucial for the transformation of stored fatty into soluble sugars for germination energy requirements, with some DEGs enriched in the germination stage [38]. The genes related to substance metabolism (glycerolipid metabolism, fatty acid degradation, and starch and sucrose metabolism) and energy supply pathways (pentose phosphate pathway, glycolysis pathway, pyruvate metabolism, TCA cycle, and oxidative phosphorylation) were differentially expressed in *C. migao* seeds during germination [39]. In this study, the seed dormancy release in *A. tsaoko* was associated with starch and sucrose metabolism, glycolysis/gluconeogenesis, citrate cycle (TCA cycle) and fatty acid metabolism, which are essential for storing and providing energy sources (starch, fatty, and proteins) that contribute to the release of seed dormancy. The genes related to storage and energy pathway (starch and sucrose metabolism, glycolysis/gluconeogenesis, TCA cycle and fatty acid metabolism) were differentially expressed in transcriptome and proteome (Fig. S2, Tables 2 and 4). Some proteins, such as BGLU6, PDC2, and PLDBETA1 were up-regulated in the proteome (Table 2), suggesting that they may play a significant role in maintaining seed vigor and germination conversion ability.

## Transcription factors play a pivotal role in ***A. tsaoko*** seed dormancy release progression

Previous studies have concluded that cold stratification induces pear seed dormancy release process by activating many TFs, including ERF, MYB, bHLH, NAC, WRKY, and MADS genes [6]. Additionally, bHLH, bZIP, NAC, WRKY, and MYB genes affect the seed chilling response and act as activators or repressors to regulate the seed dormancy status [40, 41]. In this study, a plethora of TF genes are differentially expressed in transcriptome and proteome during the dormancy release process by warm stratification (e.g., ARF, bHLH, bZIP, MYB, SBP and WRKY genes) (Fig. S3, Tables 2 and 4). ARF10 and ARF16, auxin response factors, control the expression of ABI3 during seed germination in *Arabidopsis*, suggesting ARF plays vital roles in plant response to chilling and the transition from seed/bud dormancy to germination by coordinated regulation of IAA and ABA signaling [35]. The bHLH plays multifaceted roles in ABA biosynthesis and signaling pathways. bHLH57 positively regulates the expression of ABA biosynthetic enzyme genes NCED6 and NCED9 and leads to higher ABA levels to induce seed dormancy [42]. ICE1(INDUCER OF CBF EXPRESSION1 ), a bHLH TF with a vital role in modulating the plant temperature response, interacts with ABI5 and DELLA protein to fine-turn ABA signaling suppressing seed germination [43]. Here, 5 bHLH (Unigene24037_At_Mix, CL8841.Contig1_At_Mix, Unigene8886_At_Mix, Unigene8888_At_Mix and Unigene8889_At_Mix) was identified with different expression profiles during the seed dormancy release process by warm stratification (Fig. S3a), indicating its potential role in regulating the seed dormancy release of *A. tsaoko*. The overexpression of MYB330 in tobacco reduced the expression of the ABA catabolic gene and gibberellin biosynthesis genes, affecting ABA-GA crosstalk to inhibit seed germination [44]. A homologous of MYB44 (Gene.43178_Unigene6226_At_Mix) was identified with up-regulated expression profiles during the middle and late stage of seed dormancy release (Table 2), indicating MYB44 positively regulates the seed dormancy release of *A. tsaoko*. ABI4 plays multifaceted roles in hormone signaling and metabolite pathways to regulate seed dormancy and germination, including SA, ABA, GA, starch and fatty acid metabolism [45–49]. According to the transcriptome and proteome data, different families’ TFs have diverse expression profiles in response to warm stratification and might have regulatory functions in seed dormancy release regulation.

## Changes in cell wall proteins, heat shock proteins, seed dormancy and germination proteins during ***A. tsaoko*** seed dormancy release progression

The cell wall protein, heat shock proteins, seed dormancy and germination proteins were identified in the transcriptome and the proteome data, suggesting that multiple functional regulatory proteins are involved in the regulation of seed dormancy release in *A. tsaoko* (Fig. S3, Tables 2 and 4). Cell wall proteins have been assumed to be involved in the regulation of seed germination, which contributes to the cell wall disassembly associated with endosperm cap weakening, including cellulases (CEL), mannanases (MAN), xyloglucan endotransglycosylases/hydrolases (XTH), pectin lyases (PLs), pectin methylesterases (PME), polygalacturonases (PG) and expansin (EXP) [50, 51]. Previous studies indicated that EXP was involved in GA and ABA-mediated seed dormancy release [52–54]. Based on the proteome analysis, we found that XTH7 and EXPA4 proteins were significantly up-regulated during the dormancy release process by warm stratification, suggesting potential connections between seed dormancy release and changes in cell wall function (Table 2). Heat shock proteins, a stress-responsive family of proteins, are involved in posttranslational modifications, protein folding, aggregation and disaggregation. Previous studies have concluded that *GhHSP24.7* controls seed germination in a temperature-dependently manner [55], and HSFA6b operates as a downstream regulator of the ABA-mediated stress and heat response [56]. In proteome data, HSP80 and HSP81 proteins showed an obvious down-regulation at the seed dormancy release process (Table 2), which is consistent with changes in ABA content, suggesting HSP protein potentially connects ABA signaling and ABA-mediated heat responses to regulate seed dormancy release. A group of seed dormancy and germination-specific genes and proteins have been identified in seed development [57]. In *Arabidopsis*, ASPG1(ASPARTIC PROTEASE IN GUARD CELL 1) plays an important role in the degradation of seed storage proteins during the process of dormancy, viability, and germination, which can regulation of GA signaling to seed germination [58]. In this study, 2 AGPS2 proteins were found to be expressed during the process of seed dormancy release. Therefore, we speculate that ASPG2 may regulate the seed dormancy release of *A. tsaoko* toward warm stratification, but this needs to be further confirmed.

## Regulatory networks associated with ***A. tsaoko*** seed dormancy release progression in warm stratification

**Fig. 7:**
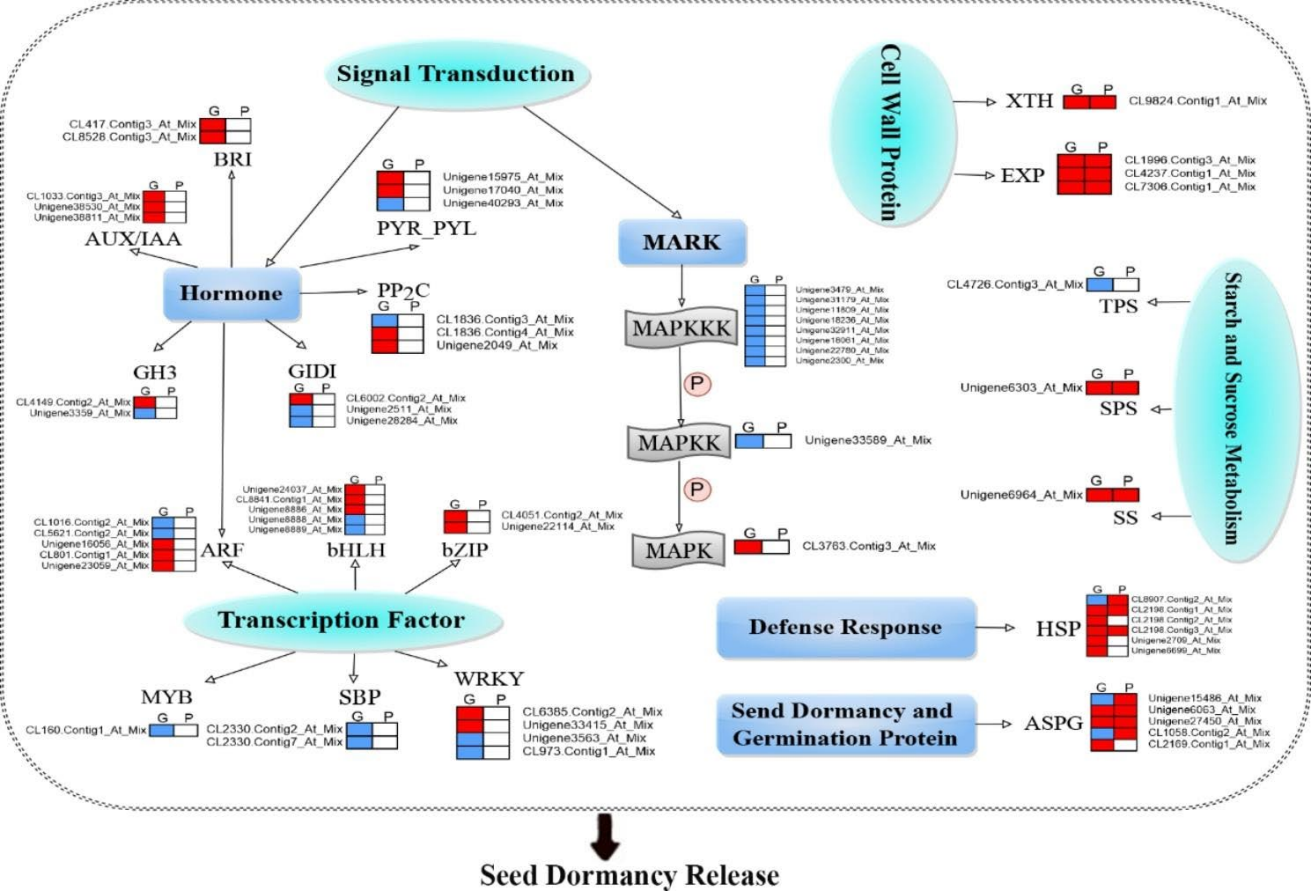
A proposed model of the regulatory network associated with *A. tsaoko *seed dormancy release progression in warm stratification. G and P in the box above represent gene and protein expression, respectively. The red box represents upregulated genes/proteins that are under the late period of seed dormancy release (S90) compared with the control (CK), and the blue box represents downregulated genes/proteins. White boxes indicate that the genes/proteins have no obvious expression change

Seed dormancy release is controlled by various regulatory processes, including signal transduction pathways (MAPK signaling, hormone) and metabolism processes (cell wall, storage and energy reserves) [20, 21]. In this study, based on transcriptome and proteome analysis, a putative model of the regulatory network associated with *A. tsaoko* seed dormancy release progression in warm stratification was proposed, in which the expression patterns of the related DEGs or DEPs were also exhibited (Fig. 7). MAPKKK and MAPKK were downregulated at the gene level, while the upregulation of MAPK in the MAPK signal pathway. Most of the hormone signal-related genes, such as PP2C, PYR/PYL, GID1, GH3, ARF, AUX/IAA and BRI genes were upregulated at the gene level. Transcription factors ARF, bHLH, bZIP, MYB, SBP and WRKY are involved in the seed dormancy release process by warm stratification. In addition to the upregulation of bZIP, the genes MYB were all downregulated, and the genes bHLH, WRKY, and ARF were partially downregulated. In starch and sucrose metabolism, 3 genes and proteins were involved in this pathway. Both SS and SPS were upregulated in the gene and protein levels, except for the TPS, which was downregulated in the gene level. 4 genes and proteins were involved in cell division, cell expansion, and cell cycles, and all XTH and EXP were upregulated. However, in the heat shock proteins and seed dormancy and germination proteins, most HSP and ASPG were differentially expressed in the seed dormancy release progression, but some of their expression patterns were opposite in gene and protein levels. Therefore, the variation of DEGs/DEPs is very complex and irregular under warm stratification during *A. tsaoko* seed dormancy release, which provides a basis for building better network-based molecular models in further research. Taken together, the result suggested that these genes and proteins may play a crucial role in the regulatory network of *A. tsaoko* seed dormancy release progression by warm stratification.

## Conclusion

3196 differentially expressed genes and 1414 differentially expressed proteins were identified to be involved in the regulation of *A. tsaoko* seed dormancy release by warm stratification. These results reveal the synergy of genes/proteins related to signal transduction pathways (MAPK signaling, hormone,) and metabolism processes (cell wall, storage and energy reserves) in the processes of seed dormancy release. A hypothetical model of the genetic regulatory network was proposed to strengthen our understanding of the mechanism of low germination rate in *A. tsaoko* at the molecular level and provides a theoretical basis for overcoming the physiological dormancy in *A. tsaoko* in the future.

## Materials and methods

### Seed preparation

The freshly mature seeds of *Amomum tsaoko* were collected from 10-year-old plants from a plantation in Napo County, Guangxi province, China (101 N, 113E). After mixing with moist perlite (volume ratio 1:3), seeds were stored in temperature-controlled incubators at 15 ℃ as a warm stratification [2]. To test seed germination, perlite was used as medium and the incubator was set to 20/30 ℃ (night/day) with a 12 h light/12 h dark cycle as reported in our previous study [2]. Germination capacity was recorded after 28 days of the experiment and the seed germination rate was calculated as the ratio of the number of seeds with the radicle protruding from the seed coat to the total number of seeds tested. There were three replicates per treatment with 100 seeds per germination box. Seeds were collected for transcriptome and proteome analysis after warm stratification of 30d (S30, early seed dormancy release period), 60d (S60, middle seed dormancy release period) and 90d (S90, late seed dormancy release period), respectively. The fresh seeds without stratification treatment were considered as the control (CK). Embryos were stripped with sterilized blades, frozen in liquid nitrogen immediately and stored at -80 ℃.

### Quantitation of endogenous hormone and observation of cell ultrastructure

Endogenous hormones of seeds (embryo) with different dormancy release statuses were determined by a UHPLC-MS/MS system. The embryos separated from seeds with a sterile scalpel were extracted with 75% methanol containing 5% formic acid. UHPLC-MS/MS analysis was performed using Ultra Performance Liquid Chromatography (Shim-pack UFLC SHIMADZU CBM30A) coupled with tandem mass spectrometry (Applied Biosystems 6500 Quadrupole Trap). The mobile phase A, consisting of 0.05% formic acid in the water, and the mobile phase B, consisting of 0.05% formic acid in the acetonitrile, was used for chromatographic separation by the Agilent C18 column. An electrospray ionization (ESI) mode was used to obtain high-resolution mass spectra (HRMS). Three biological independent replicates were performed.

The cell ultrastructure of seed embryos was investigated using transmission electron microscopic (Talos L120C G2, Thermo Fisher Scientific). Seed embryos were fixed in 2.5% glutaraldehyde in 0.1 M PBS (phosphate buffer solution). Subsequently, the samples were washed four times with 0.1 M PBS and 1% osmic acid, respectively. And then, the samples were dehydrated with graded 30–70% ethanol series concentrations and graded 70–100% acetone series concentrations, respectively. The samples were infiltrated and embedded in Spurr’s resin, and 80 nm ultrathin sections were obtained by ultramicrotome (LEICA EM UC7) and observed in a transmission electron microscope. Three biological independent replicates were performed.

### RNA extraction and Illumina sequencing

Since 30d, 60d and 90d of warm stratification represented critical time points of seed dormancy release of *A. tsaoko* as reported in our previous study [18], the embryos of CK, S30, S60 and S90 were used in RNA extraction and transcriptome profiling analysis. To prepare Illumina RNA-seq libraries, the total RNA of 12 embryo samples from CK, S30, S60, and S90 (three replicates per dormancy release stage) were extracted using Takara MiniBEST Universal RNA Extraction Kit (Beijing, China) by the manufacturer’s instructions. The purity, concentration and integrity of each RNA sample were validated using 1% agarose electrophoresis, Nanodrop 2000 microspectrophotometer and Agilent 2100 Bioanalyzer, respectively. For RNA-seq libraries construction, RNA samples (RIN>7.5) were enriched by Oligo-dT magnetic beads. The purified RNA was fragmented into short pieces by fragment buffer and then the reverse transcription was carried out with N6 primer. The double-strand cDNA was end-repaired, adaptor-ligated, and followed by PCR amplification. The amplified PCR product was heat-denatured into single-stranded DNA, single-stranded DNA was then circularized to make a single-stranded circular DNA library. Finally, paired-end sequencing of the cDNA library was performed on the BGISEQ-500 platform.

### Transcriptome profiling and DEGs analysis

After sequencing, raw sequence data were filtered by removing low-quality reads (bases with a mass value less than 15, accounting for over 20% of the total bases) and reads with an adaptor. De novo assembly of clean reads was performed by Trinity software, and this was followed by the operation of cluster and de-redundant using Tgicl software. Then, seven functional databases (NT, NR, GO, KOG, KEGG, SwissProt and InterPro) were used for annotation analysis. The expression levels for all transcripts were quantified by calculating FPKM (fragments per kilobase of transcript per million mapped reads) as described by Trapnell [59]. The differential expressed genes (DEGs) were screened with a threshold of |log2 (fold change) |>1 and a statistical significance of p-value <0.05 with the NOISeq method [60]. Gene ontology (GO) enrichment of DEGs was performed with Gene Ontology (http://www.geneontology.org/), and KEGG pathway enrichment of DEGs was performed using the KEGG database (http://www.kegg.jp/) [61–63], a corrected p-value ≤ 0.05 as the threshold and rich factor.

### Protein extraction and TMT analysis

12 embryo samples (three biological replicates for each stratification stage) were grounded into powder respectively with liquid nitrogen. Four volumes of lysis buffer (8 M urea, 1% TritonX-100, 10 mM dithiothreitol and 1% Protease Inhibitor Cocktail) were added to the powder. After sonication by a high-intensity ultrasonic processor, the cell debris was removed by centrifugation at 12,000 rpm, 4 ℃ for 10 min. After precipitation with 20% TCA for 2 h at -20 ℃ and centrifugation at 12,000 rpm, 4 ℃ for 10 min, protein precipitate was collected and washed with pre-cooled acetone three times, and then redissolved in 8 M urea. Protein concentration was determined by the BCA method. The protein solution was added with dithiothreitol to a final concentration of 5 mM for reduced reaction at 56 ℃ for 30 min and with iodoacetamide to a final concentration of 0.11 mM for alkylated reaction at room temperature for 15 min in the dark and then digested with trypsin (Promega, Madison, WI, USA) (enzyme: protein ratio of 1:50) at 37 °C for 12 h. Desalted peptides were labeled with TMT reagents (TMT Mass Tagging Kit and Reagents, Thermo, Foster, CA, USA), as instructed by the manufacturer. The labeled peptide mix was fractionated using a C18 column (Agilent 300Extend C18 4.6 × 250 mm, 5 μm) on a nano-liquid chromatography-MS/MS (LC-MS/MS) by the Q Exactive system (Metware, Wuhan, China). Peptides were separated online by reverse phase chromatography by using 8% (vol/vol) to 32% (vol/vol) acetonitrile in a 60 min gradient. The Q Exactive was operated in the data-dependent mode with the following settings: 70,000 resolution, 350–1800 m/z full scan, and a 100 m/z isolation window.

### Proteomic profiling and DEPs analysis

Identification of peptides was done with MaxQuant 1.5.2.8 by using a 1% false discovery rate (FDR) against the protein database. Peptides were searched for variable modification of N-term protein acetylation, oxidation (M), and deamidation (NQ), with a 5 ppm mass error and a mass tolerance for fragment ions set as 0.02 Da. Proteins with a P < 0.05 and a fold change > 1.50 or < 0.67 were defined as differentially expressed proteins (DEPs). These proteins were classified and grouped using the GO and KEGG databases [61–63]. The interaction network and subcellular location were predicted using the STRING protein interaction database and WoLF PSORT software. Domain information was obtained from the Pfam database.

### Correlation between transcript and protein

The Pearson correlation coefficient (r) was calculated to evaluate the concordance between transcriptome and proteome profiles according to the fold change of expressed transcripts and proteins. GO and KEGG enrichment analyses for DEGs-DEPs were then conducted [61–63].

### qRT-PCR analysis

50 DEGs were selected for qRT-PCR analysis, and *A. tsaoko UBCE2* was used as an internal control [19]. The primers were designed based on the sequence of transcripts using Primer 5.0 and are shown in Table S8. The total RNA of embryo samples from CK, S30, S60, and S90 were extracted and reverse-transcribed into cDNA. One-third dilution of the cDNA sample was assessed by quantitative real-time PCR using the CFX96 instrument (Bio-Rad Company, USA). All of the experiments possessed three biological replicates with three technical replicates for each reaction. The 2^–ΔΔCt^ method was used to estimate the relative expression level. The normalized values of relative expression and FPKM values were calculated using log2 fold variation measurements, and the correlation between RNA-seq and qPCR results was analyzed using these values.

## Electronic supplementary material

Below is the link to the electronic supplementary material.

**Additional file**1: **Table S1**. Data information of *A.tsaoko* transcriptome. **Table S2**. The differentially expressed genes shared in three comparison. **Table S3**. The differentially expressed genes involved in signal transduction pathways. **Table S4**. The detailed information of DEPs. **Table S5**. Gene ontology term assignments for the DEPs. **Table S6**. The detailed information of proteins correlated with transcripts. **Table S7**. qRT-PCR analysis of 50 DEGs. **Table S8**. qRT-PCR primers for candidate genes.

**Additional file 2:** **Fig. S1**. Phenotypes of seeds and embryos for *A.tsaoko* under different warm treatment times. **Fig. S2**. Heat map analysis of DEGs in MAPK signaling pathway (a), plant hormone signal transduction (b), starch and sucrose metabolism (c), and fatty acid biosynthesis (d). Red and blue represent increased and decreased transcript abundance, respectively. **Fig S3**. Heat map analysis of DEGs in transcription factors (a), cell wall (b), and heat shock protein (c). Red and blue represent increased and decreased transcript abundance, respectively. **Fig. S4**. KEGG pathway annotation of the DEPs. **Fig. S5**. Distribution of quantitative Pearson’s correlation coefficients between transcriptome and proteome. **Fig. S6**. Correlation plot of the RNA-seq results and qRT-PCR results. Results were calculated using log2 fold variation measurements.

## Data Availability

The raw sequencing data have been submitted to the NCBI SRA database (PRJNA869478, https://www.ncbi.nlm.nih.gov/sra/?term=PRJNA869478). The assembled genes data also were submitted to the GenBank TSA database (GKAY01000000, https://www.ncbi.nlm.nih.gov/nuccore/GKAY00000000.1).
