# Supplementary material for: Transcriptome and proteome analyses reveal the potential mechanism of seed dormancy release in Amomum tsaoko during warm stratification
Source: BMC Genomics. 2023 Mar 2;24:99. doi: 10.1186/s12864-023-09202-x (PMC9983222; doi:10.1186/s12864-023-09202-x)
Supplement: Supplementary file 2 — Additional file 2: Fig. S1. Phenotypes of seeds and embryos for A.tsaoko under different warm treatment times. Fig. S2. Heat map analysis of DEGs in MAPK signaling pathway (a), plant hormone signal transduction (b), starch and sucrose metabolism (c), and fatty acid biosynthesis (d). Red and blue represent increased and decreased transcript abundance, respectively. Fig S3. Heat map analysis of DEGs in transcription factors (a), cell wall (b), and heat shock protein (c). Red and blue represent increased and decreased transcript abundance, respectively. Fig. S4. KEGG pathway annotation of the DEPs. Fig. S5. Distribution of quantitative Pearson’s correlation coefficients between transcriptome and proteome. Fig. S6. Correlation plot of the RNA-seq results and qRT-PCR results. Results were calculated using log2 fold variation measurements. [file 12864_2023_9202_MOESM2_ESM.docx]

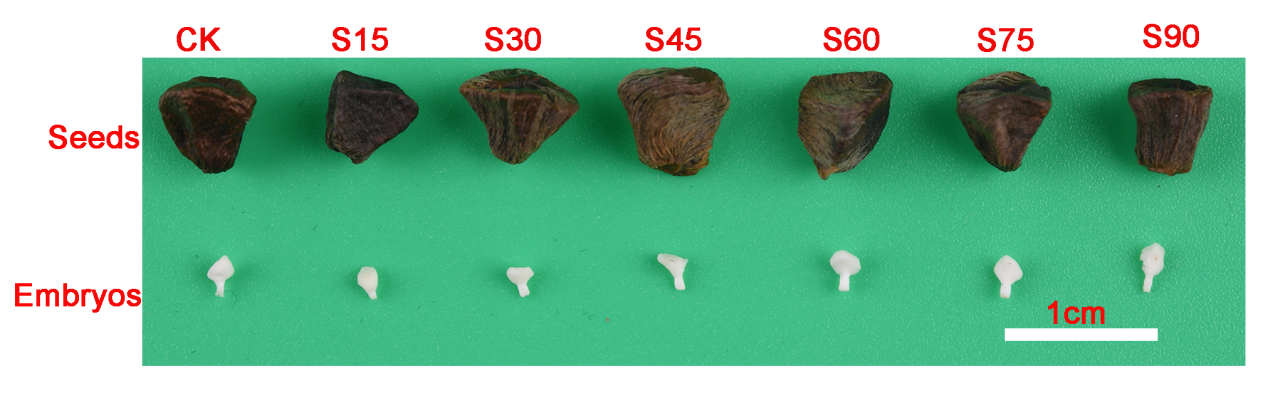


**Fig. S1 Phenotypes of seeds and embryos for *A.tsaoko* under different warm treatment times.**


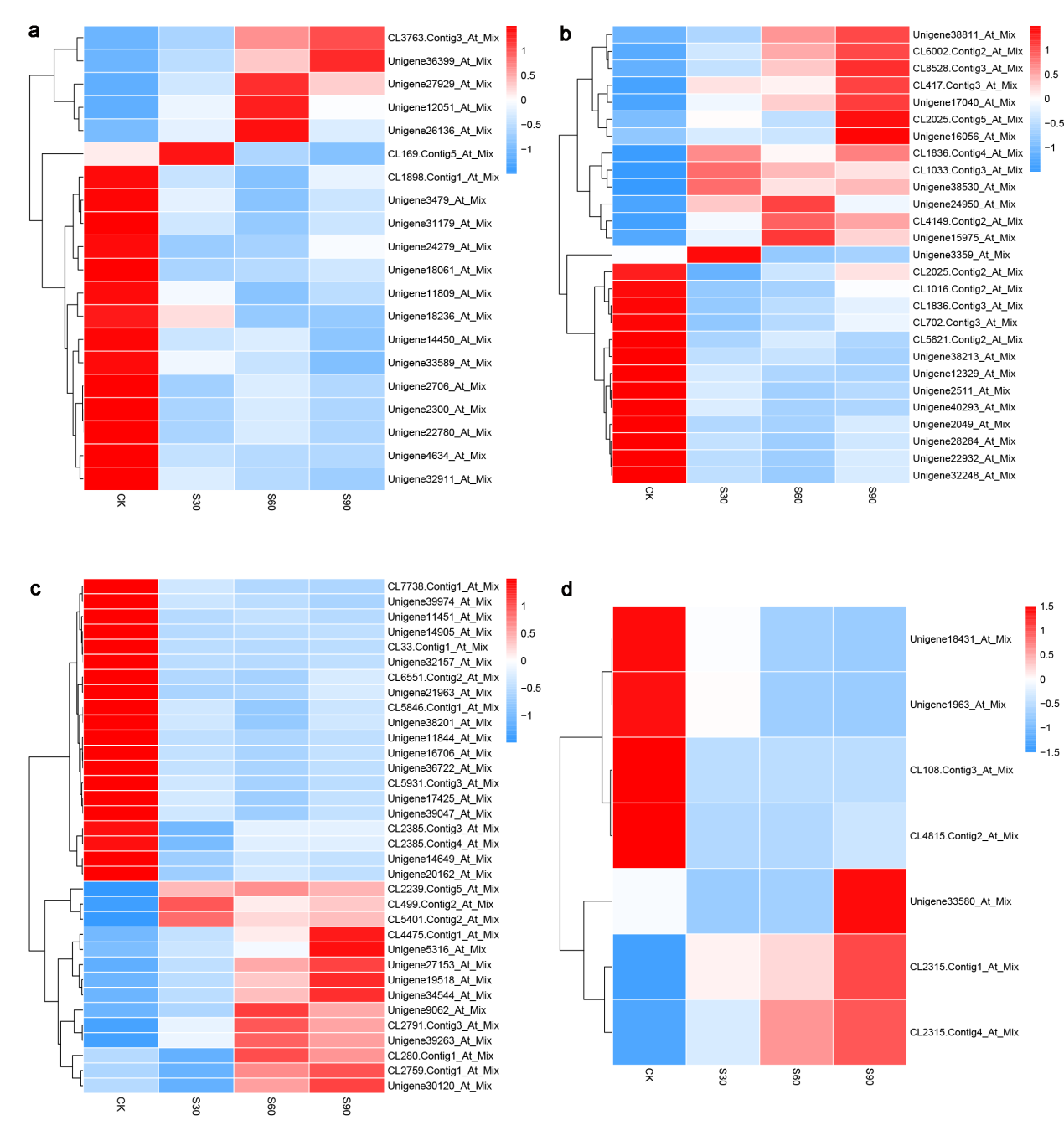


**Fig. S2 Heat map analysis of DEGs in MAPK signaling pathway (a), plant hormone signal transduction (b), starch and sucrose metabolism (c), and fatty acid biosynthesis (d**). Red and blue represent increased and decreased transcript abundance, respectively.


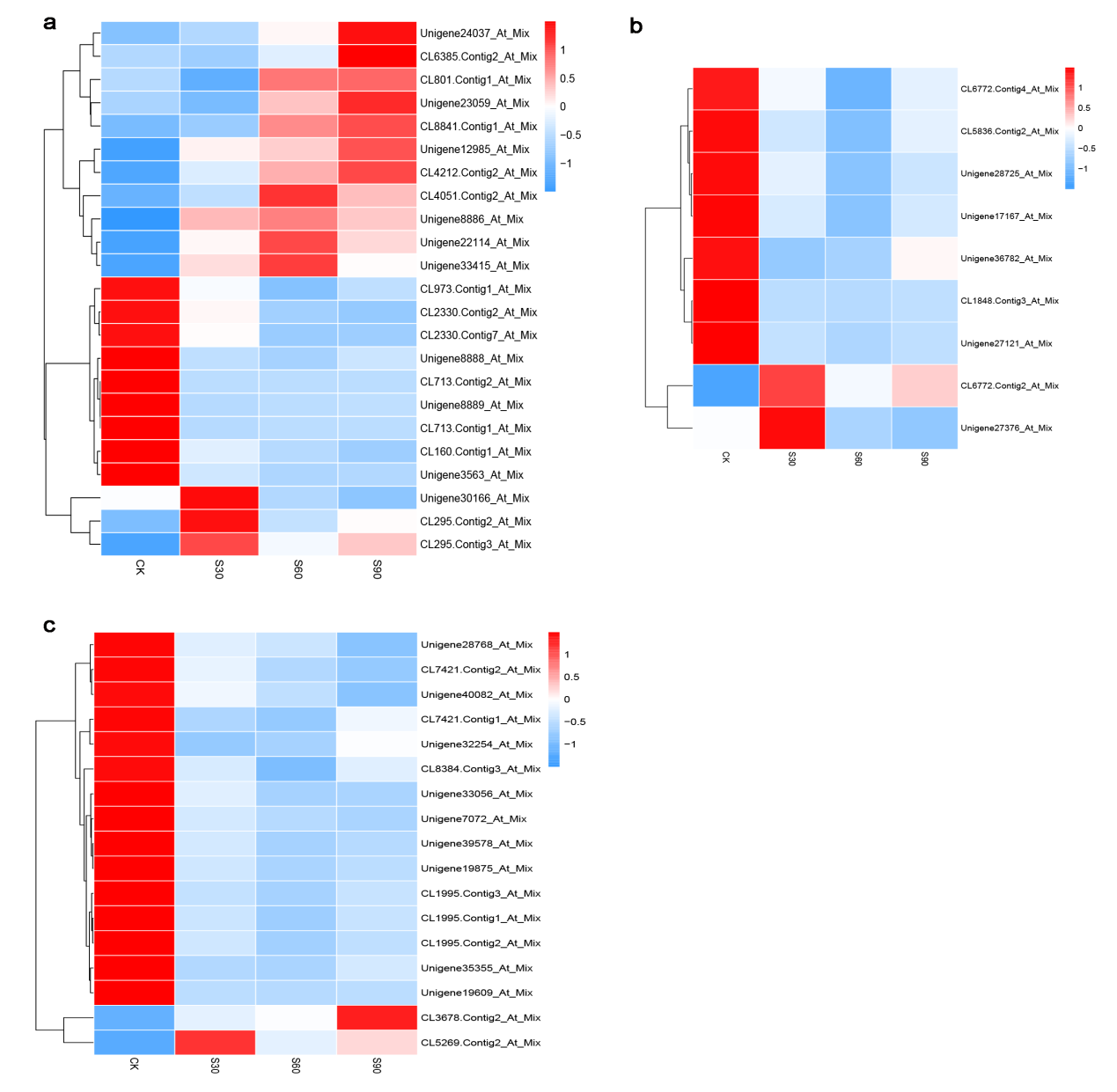


**Fig.S3 Heat map analysis of DEGs in** **transcription factors (a), cell wall (b), and heat shock protein (c).** Red and blue represent increased and decreased transcript abundance, respectively.


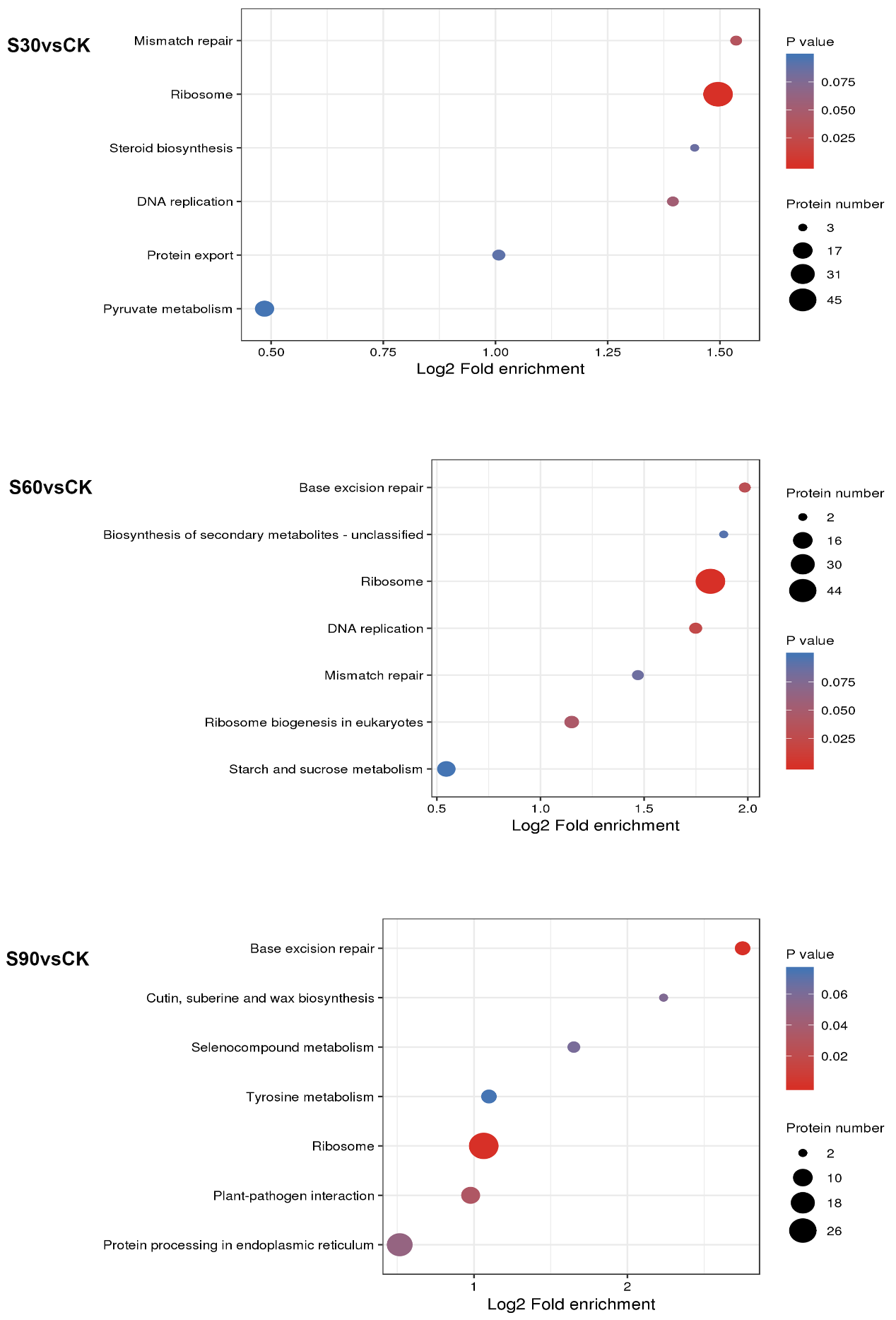


**Fig. S4 KEGG pathway annotation of the DEPs.**


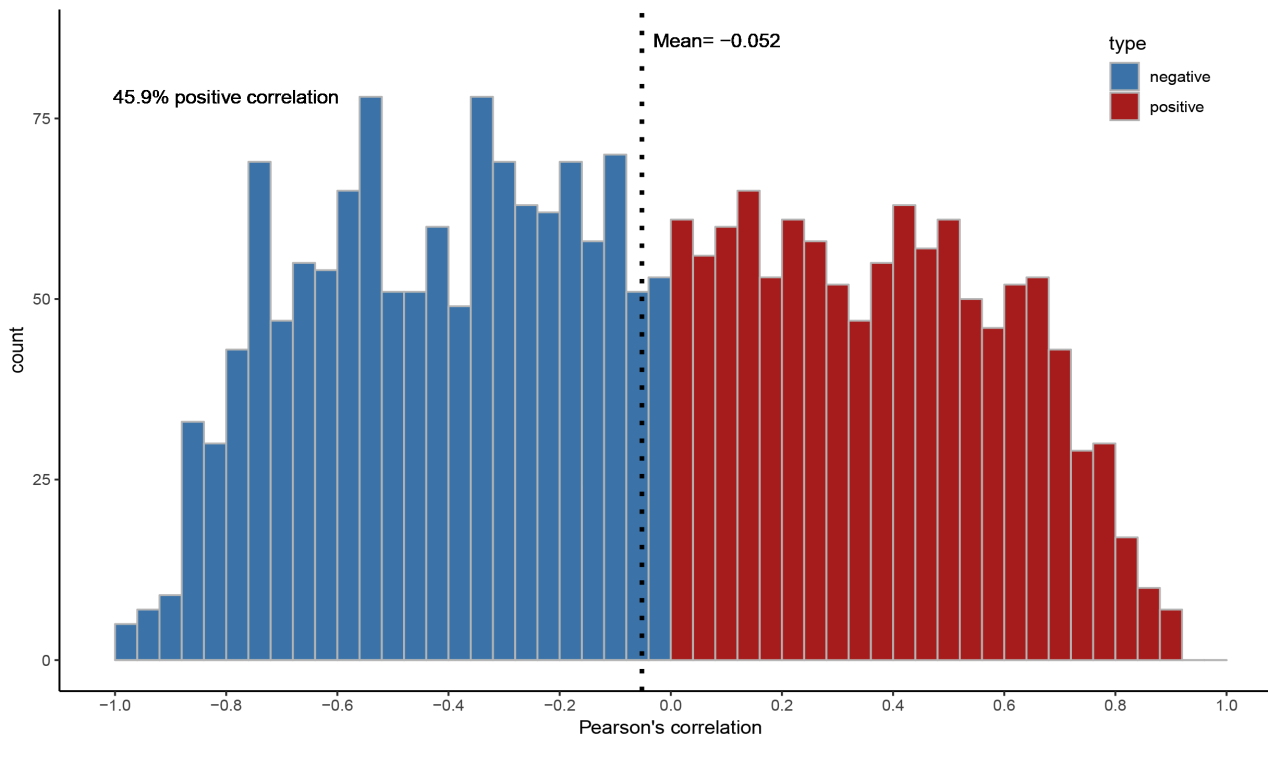
**Fig. S5 Distribution of quantitative Pearson's correlation coefficients between transcriptome and proteome.**

**
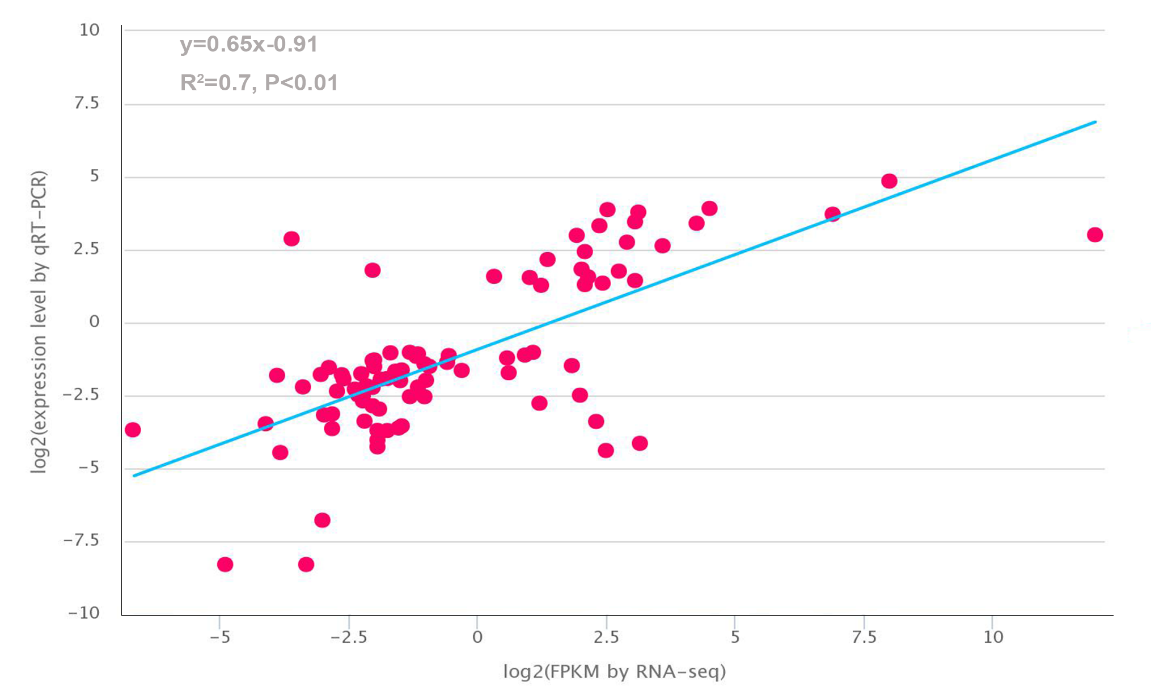
**

**Fig. S6 Correlation plot of the RNA-seq results and qRT-PCR results. Results were calculated using log2 fold variation measurements.**
